# Factors affecting the urinary aldosterone-to-creatinine ratio in healthy dogs and dogs with naturally occurring myxomatous mitral valve disease

**DOI:** 10.1186/s12917-020-02716-6

**Published:** 2021-01-07

**Authors:** Alberto Galizzi, Mara Bagardi, Angelica Stranieri, Anna Maria Zanaboni, Dario Malchiodi, Vitaliano Borromeo, Paola Giuseppina Brambilla, Chiara Locatelli

**Affiliations:** 1 Department of Veterinary Medicine, University of Milan, Via dell’Università 6, 26900 Lodi, Italy; 2 Department of Computer Science & Data Science Research Center, University of Milan, Milan, Italy

**Keywords:** Dog, MMVD, Aldosterone, UAldo:C, RAAS, LA/Ao

## Abstract

**Background:**

Chronic renin-angiotensin-aldosterone system (RAAS) activation in course of heart diseases contributes to cardiac remodeling and heart failure. Myxomatous mitral valve disease (MMVD) is characterized by different stages of severity and trend of RAAS activity during the course of the disease is still uncertain. The urinary aldosterone-to-creatinine ratio (UAldo:C) has been proven to reflect RAAS activation in dogs and might be a useful marker in monitoring therapy and disease progression, but data about this parameter need to be expanded. The objective of this study was to evaluate the UAldo:C in healthy dogs and dogs with naturally occurring MMVD, and to investigate the relationships between this parameter and clinical, echocardiographic and laboratory variables.

**Results:**

The study population consisted of 149 dogs: 49 healthy and 100 MMVD dogs (45 stage B1, 13 stage B2 and 42 stage C). Urinary aldosterone-to-creatinine ratio was not significantly different among healthy and MMVD dogs of any stages. Breed, sex and age showed a significant impact on UAldo:C. In particular, Chihuahua and Cavalier King Charles spaniel showed significantly higher UAldo:C than other breeds, as well as intact females than other genders. In stage C dogs, UAldo:C appeared to be increased by spironolactone and was positively associated with furosemide dose (*P* = 0.024). Aldosterone breakthrough (ABT) appeared to occur in 36% (8/22) of stage C dogs not receiving spironolactone. A significant positive association between UAldo:C and left atrium-to-aortic root ratio (LA/Ao) was found.

**Conclusions:**

Individual factors such as breed, sex and age appeared to influence UAldo:C, and therapy seemed to add further variability. In the light of these results, comparing the UAldo:C of a single patient with a population-based reference value might lead to wrong interpretations and an individual monitoring should be considered. The prevalence of ABT in the present study (36%) was in line with those previously reported. However, due to the high individual variability of UAldo:C found in the study, even this result should be re-evaluated in the setting of an individual longitudinal approach. The positive association between UAldo:C and LA/Ao supports the mutual relationship between RAAS and cardiac remodeling.

## Background

The renin angiotensin aldosterone system (RAAS) represents an important compensatory mechanism of heart failure. However, a chronic activation is maladaptive and contributes to the development of cardiovascular remodeling and congestive pattern because of the harmful cardiovascular and renal effects of angiotensin II (A-II) and aldosterone [1–8]. Indeed, higher aldosterone levels have been associated with cardiac remodeling and worse outcome in humans with heart diseases [6, 9–16], and urinary aldosterone (UAldo) concentration has appeared to be associated with greater ventricular remodeling and a worse prognosis in dogs with myxomatous mitral valve disease (MMVD) [17, 18]. Moreover, the beneficial effects of angiotensin-converting enzyme inhibitors (ACEI) and spironolactone in patients with congestive heart failure (CHF) have been showed in both species, indirectly proving the negative impact of chronic RAAS stimulation [1, 19–22].

The MMVD is the most common acquired cardiovascular disease in dogs. Due to its chronic and progressive nature, MMVD is characterized by different stages of severity, ranging from a pre-clinical phase (stage B1 and B2, American College of Veterinary Internal Medicine [ACVIM] classification) to the onset of CHF and related clinical signs (stage C and D) [23]. The trend of RAAS activity during the course of the disease is still uncertain. While it is fairly established that RAAS is overstimulated after the onset of CHF secondary to various heart diseases [2, 24–26], there are conflicting data about the neurohormonal activation during the asymptomatic phase [2, 17, 27–36]. Accordingly, whereas the administration of RAAS blockers (eg, ACEI, spironolactone) is recommended in stage C and D, their use in pre-clinical MMVD is still subject of debate [1, 23]. Moreover, the aldosterone breakthrough (ABT) phenomenon suggests the possibility of RAAS overexpression even after the beginning of ACEI therapy [37, 38].

Therefore, the assessment of RAAS activity in course of MMVD could help optimize the follow-up and the therapeutic management of the patient. The urinary aldosterone-to-creatinine ratio (UAldo:C) seems to be a very useful parameter for the monitoring of RAAS activity in the clinical practice. It has been proven to reflect RAAS activation and to be comparable to 24 h urinary aldosterone excretion, which, unlike serum/plasma aldosterone, is not affected by the pulsatile variations of aldosterone secretion. Secondly, it can be easily determined from a single “free-catch” urine sample, thus avoiding blood sampling and reducing the impact of stress of in-hospital visit [38–40]. Moreover, compared to other RAAS components, aldosterone has the advantage of being the last effector of the cascade; thus, its assessment also takes into account the alternative pathways of RAAS, such as the angiotensin-converting enzyme (ACE) or A-II independent ones [1]. However, to our knowledge, data about UAldo:C in healthy and MMVD populations are still not enough consolidated and should be expanded before introducing it in the diagnostic routine. The first aim of this study was to evaluate the UAldo:C in healthy dogs and dogs with naturally occurring MMVD in stage B1, B2 and C, in order to assess their RAAS activity. A second aim of the study was to investigate the relationships between UAldo:C and certain clinical, echocardiographic and laboratory variables.

## Results

### Animals

The study population consisted of 149 dogs of which 49 were healthy, 45 were stage B1, 13 were stage B2 and 42 were stage C. Demographic data for all of the dogs included and for the different groups are reported in Table 1.

**Table 1:** Demographic data in all dogs, healthy dogs and dogs with different stages of MMVD

	All dogs	Healthy	Stage B1	Stage B2	Stage C
Number	149	49	45	13	42
Age (years)	9 (6–12.25)	6 (3–7.50) ^†‡§^	9.99 ± 3.37	9 (8–12)	11.71 ± 2.88
Weight (kg)	9.40 (6.18–17.25)	16 (7.33–27.90) ^§^	9.40 (7.45–18.15)	8.6 (3.08–11.75)	7 (4.95–10.25)
Sex (F/M)	75/74	30/19	20/25	6/7	19/23
Neuter status (IF/NF/IM/NM)	23/52/52/22	16/14/13/6 ^†§^	3/17/16/9	1/5/6/1	3/16/17/6
Breed		†‡§	§		
CKCS	27	6	8	6	7
CHH	18	7	4	3	4
JRT	8	3	4	0	1
Other breeds < 15 kg	53	6	16	3	28
Other breeds ≥15 kg	43	27	13	1	2

*CKCS* Cavalier King Charles Spaniel, *CHH* Chihuahua, *JRT* Jack Russell Terrier, *F* Females, *M* Males, *IF* Intact Females, *NF* Neutered Females, *IM* Intact Males, *NM* Neutered Males. Other breeds < 15 kg**:** 22 crossbreed (1 healthy, 8 stage B1, 2 stage B2, 11 stage C), 6 Dachshund (2 stage B1, 4 stage C), 5 Miniature Pinscher (1 stage B2, 4 stage C), 4 Toy Poodle (2 healthy, 2 stage B1), 4 Maltese (4 stage C), 3 Pug (2 healthy, 1 stage B1), 2 Shih-tzu (1 stage B1, 1 stage C), 1 Bichon Frisé (1 healthy), 1 Chinese Crested Dog (1 stage C), 1 English Cocker Spaniel (1 stage C), 1 Fox Terrier (1 stage C), 1 Bruxelles Griffon (1 stage B1), 1 Pekingese (1 stage C), 1 Yorkshire Terrier (1 stage B1). Other breeds ≥15 kg: 15 crossbreed (6 healthy, 7 stage B1, 1 stage B2, 1 stage C), 4 American Staffordshire Terrier (4 healthy), 4 Golden Retriever (4 healthy), 3 Pointer (2 healthy, 1 stage B1), 2 German Shepherd (1 healthy, 1 stage B1), 2 Staffordshire Bull Terrier (2 healthy), 1 Great Dane (1 healthy), 1 Standard Poodle (1 healthy), 1 Border Collie (1 stage B1), 1 Boxer (1 stage B1), 1 Cane Corso (1 stage B1), 1 Drahthaar (1 stage C), 1 Labrador Retriever (1 healthy), 1 Belgian Shepherd (1 healthy), 1 Rhodesian Ridgeback (1 healthy), 1 Standard Schnauzer (1 healthy), 1 English Setter (1 stage B1), 1 English Springer Spaniel (1 healthy), 1 Italian Hound (1 healthy)

Data are reported as mean ± standard deviation for normally distributed variables and median (interquartile range) for non-normally distributed variables

†values significantly differ (*p* < 0.05) from stage B1; ‡values significantly differ (*p* < 0.05) from stage B2; §values significantly differ (*p* < 0.05) from stage C

Age was significantly lower in healthy dogs compared to stage B1, B2 and C dogs (*P* values < 0.001). Body weight (BW) was significantly higher in healthy dogs compared to stage C dogs (*P* values < 0.05). No significant differences were detected in age and BW between any other group pairs. There were no significant differences by sex among the four groups. Neuter status distribution was significantly different for healthy dogs compared to stage B1 and stage C dogs (*P* values < 0.05). Breed distribution was significantly different for healthy dogs compared to stage B1, B2 (*P* values < 0.05) and C dogs (*P* value ≤0.001), as well as between stage B1 and stage C dogs (*P* value < 0.05). No significant differences were detected in neuter status and breed distributions between any other group pairs.

At the time of enrolment, 9/13 stage B2 dogs were already receiving pimobendan. All 42 stage C dogs were already receiving standard therapy (furosemide, ACEI, pimobendan) and 20 out of these were also treated with spironolactone. For stage C dogs, the median (interquartile range [IQR]) dosages of furosemide and ACEI were 2.93 (2–5) and 0.58 (0.39–0.82) mg/kg/day, respectively; the median (IQR) durations of furosemide and ACEI administration were 307.5 (46–579.75) and 344 (47–959) days, respectively. The median (IQR) dosage and duration of spironolactone administration (20/42 stage C) were 2.75 (2.23–2.97) mg/kg/day and 248 (70–488.5) days, respectively. Nine stage B2 and all stage C were receiving pimobendan at a standard dose of 0.25–0.30 mg/kg q12h.

### Echocardiographic parameters

Echocardiographic parameters and systolic arterial pressure (SAP) for all of the dogs included and for the different groups are shown in Table 2.

**Table 2:** Echocardiographic parameters and systolic arterial pressure in all dogs, healthy dogs and dogs with different stages of MMVD

	All dogs	Healthy	Stage B1	Stage B2	Stage C
Number	149	49	45	13	42
LA/Ao	1.42 (1.17–1.89)	1.20 (1.08–1.39) ^‡§^	1.26 (1.14–1.43) ^‡§^	1.79 ± 0.22 ^§^	2.31 (1.84–2.69)
LVEDDn	1.55 (1.35–1.86)	1.42 ± 0.18 ^‡§^	1.39 (1.27–1.63) ^‡§^	1.83 ± 0.22	2.00 ± 0.35
LVESDn	0.91 (0.78–1.05)	0.89 ± 0.15 ^§^	0.86 ± 0.18 ^§^	0.96 ± 0.14	1.04 (0.95–1.14)
E peak velocity (m/s)	0.83 (0.65–1.13)	0.67 ± 0.16 ^‡§^	0.75 ± 0.17 ^‡§^	0.97 ± 0.20 ^§^	1.32 ± 0.32
A peak velocity (m/s)	0.70 (0.55–0.83)	0.59 ± 0.13 ^‡§^	0.68 (0.54–0.82) ^§^	0.76 (0.70–0.86)	0.81 (0.70–0.97)
E/A	1.23 (1.00–1.51)	1.17 ± 0.27 ^§^	1.12 ± 0.30 ^§^	1.20 ± 0.26 ^§^	1.66 ± 0.55
SAP	142.94 ± 19.73	145 (140–150)	141.76 ± 20.62	155 (127.50–170)	140.67 ± 20.96

*LA/Ao* Left atrium-to-aortic root ratio, *E/A* E peak velocity-to-A peak velocity ratio, *LVEDDn* Normalized left ventricular end-diastolic diameter, *LVESDn* Normalized left ventricular end-systolic diameter, *SAP* Systolic arterial pressure

Data are reported as mean ± standard deviation for normally distributed variables and median (interquartile range) for non-normally distributed variables

†values significantly differ (*p* < 0.05) from stage B1; ‡values significantly differ (*p* < 0.05) from stage B2; §values significantly differ (*p* < 0.05) from stage C

There were no significant differences in any echocardiographic parameters between healthy and B1 dogs.

Left atrium-to-aortic root ratio (LA/Ao) was higher in stage B2 and C dogs compared to healthy and stage B1 dogs (*P* values < 0.001), as well as in stage C dogs compared to stage B2 dogs (*P* value < 0.05).

Stage B2 and C dogs had higher normalized left ventricular end-diastolic diameter (LVEDDn), compared to healthy (*P* values < 0.001) and stage B1 dogs (*P* values ≤0.05). Stage C dogs had higher normalized left ventricular end-systolic diameter (LVESDn), compared to healthy and B1 dogs (*P* values < 0.001), while was not significantly different for stage B2 dogs compared to healthy and stage B1 dogs. Normalized left ventricular end diastolic diameter and LVESDn were not statistically different between stage C and B2 dogs.

E peak velocity was higher in stage B2 and C dogs compared to healthy (*P* values < 0.001) and stage B1 dogs (*P* values < 0.05), as well as in stage C dogs compared to stage B2 dogs (*P* value < 0.001). A peak velocity was higher in stage B2 and C dogs compared to healthy dogs (*P* value ≤0.001) and in stage C dogs compared to stage B1 dogs (*P* value < 0.05), while was not significantly different for stage B2 dogs compared to stage B1 and stage C dogs. E peak velocity-to-A peak velocity ratio (E/A) was higher in stage C dogs compared to healthy, stage B1 (*P* values < 0.001) and stage B2 dogs (*P* value < 0.05), while it was not significantly different for stage B2 dogs compared to healthy and stage B1 dogs.

There were no significant differences in SAP among the four groups.

### Standard laboratory parameters

Standard laboratory parameters are reported in Table 3. Serum urea (UREA) was higher in stage C dogs compared to healthy dogs (*P* value < 0.05), while no significant differences were detected between any other group pairs. Serum creatinine (SCr) was not significantly different among the four groups.

**Table 3:** Laboratory parameters in all dogs, healthy dogs and dogs with different stages of MMVD

	All dogs	Healthy	Stage B1	Stage B2	Stage C
Number	149	49	45	13	42
UREA (mg/dL)	39 (31–49.28)	33 (27.93–40-50) ^§^	35.16 ± 10.60	58 ± 25.85	49 (38.68–68.04)
SCr (mg/dL)	0.92 (0.80–1.10)	0.91 ± 0.20	0.89 ± 0.18	1.04 ± 0.32	1 (0.80–1.18)
USG	1036 (1020.50–1052)	1046.98 ± 18.41 ^§^	1042.07 ± 16.16 ^§^	1039.69 ± 20.50	1019 (1011.50–1028.50)
UP/UC	0.12 (0.04–0.27)	0.07 (0.03–0.12) ^‡§^	0.15 (0.06–0.24)	0.20 (0.10–0.41)	0.25 (0.35–0.69)
UAldo:C (μg/g)	1.86 (0.88–3.77)	1.75 (0.83–4.02)	1.75 (0.73–3.13)	1.95 (0.85–4.65)	2.03 (1.16–4.85)

*UREA* Serum urea, *SCr* Serum creatinine, *USG* Urine specific gravity, *UP/UC* Urinary protein-to-creatinine ratio, *UAldo:C* Urinary aldosterone-to-creatine ratio

Data are reported as mean ± standard deviation for normally distributed variables and median (interquartile range) for non-normally distributed variables

†values significantly differ (*p* < 0.05) from stage B1; ‡values significantly differ (*p* < 0.05) from stage B2; §values significantly differ (*p* < 0.05) from stage C

Urine specific gravity (USG) was lower in stage C dogs compared to healthy and stage B1 (*P* values < 0.001). Urinary protein-to-creatinine ratio (UP/UC) was significantly higher in stage B2 and C dogs compared to healthy dogs (*P* values < 0.05). No significant differences were detected in USG and UP/UC between any other group pairs.

### Urinary aldosterone assay validation

The enzyme-linked immunosorbent assay (ELISA) kit^1^ resulted appropriate for the measurement of aldosterone in dog urine after acid hydrolysis.

The ELISA standard curve in a semi-log plot was linear between 250 and 3.9 pg/mL.

Recoveries of added aldosterone were satisfactory, ranging between 93.0 and 113.1% (mean 101.0% ±8.1). Furthermore, slope of the regression was not different from unit (slope 0.97 ± 0.05; y-intercept 4.70 ± 5.42; r^2^  = 0.99).

Dilutional parallelism was also demonstrated. Dilutions of dog urine samples were compared with the dose-response curve for standard aldosterone. There was no significant difference (*P* > 0.1) between the slopes, after log transformation of the dilutions (*P* > 0.1).

The intra- and inter-assay coefficient of variation were calculated by measuring the urine aldosterone in four dogs. Aldosterone concentrations ranged between 1.22 and 13.51 ng/mL. The intra-assay coefficient of variation ranged between 8.2 and 16.6% and the inter-assay coefficient of variation between 14.2 and 21.3%.

### Urinary aldosterone-to-creatinine ratio (UAldo:C)

There were no significant differences in UAldo:C among healthy, stage B1, stage B2 and stage C dogs (Table 3). Pearson’s correlation and multiple linear regression analysis results are shown in Table 4 and Table 5 respectively.

**Table 4:** Pearson’s correlation between UAldo:C and other variables

	All dogs		Healthy + Stage B1		Stage B2		Stage C	
	Pearson’s coefficient	*P* value	Pearson’s coefficient	*P* value	Pearson’s coefficient	*P* value	Pearson’s coefficient	*P* value
UAldo:C-Sex	0.167	0.042*	0.247	0.016*	0.193	0.528	0.012	0.942
UAldo:C-Breed	0.346	< 0.001*	0.480	< 0.001*	0.257	0.396	0.109	0.494
UAldo:C-Age	− 0.233	0.004*	−0.365	< 0.001*	0.016	0.958	−0.181	0.252
UAldo:C-BW	−0.242	0.003*	−0.283	0.006*	−0.181	0.554	−0.125	0.429
UAldo:C-USG	0.274	0.001*	0.510	< 0.001*	0.449	0.124	−0.049	0.756
UAldo:C-UP/UC	0.069	0.405	0.165	0.113	−0.040	0.896	0.048	0.762
UAldo:C-UREA	0.124	0.203	0.164	0.199	0.722	0.043*	0.024	0.888
UAldo:C-SCr	0.103	0.291	0.047	0.717	0.557	0.151	0.127	0.459
UAldo:C-LA/Ao	0.154	0.061	0.056	0.590	0.624	0.023*	0.230	0.148
UAldo:C-LVEDDn	0.077	0.354	−0.092	0.377	−0.255	0.401	0.262	0.094
UAldo:C-LVESDn	0.096	0.246	−0.083	0.426	0.098	0.750	0.282	0.070
UAldo:C-E peak	0.035	0.681	−0.040	0.717	−0.259	0.417	0.095	0.550
UAldo:C-SAP	0.039	0.680	0.193	0.105	0.338	0.282	−0.342	0.065

Sex: female = 1, male = 0; Breed: Chihuahua = 1, any other breeds = 0; *BW* Body weight, *USG* Urine specific gravity, *UP/UC* Urinary protein-to-creatinine ratio, *UREA* Serum urea, *SCr* Serum creatinine, *LA/Ao* Left atrium-to-aortic root ratio, *LVEDDn* Normalized left ventricular end-diastolic diameter, *LVESDn* Normalized left ventricular end-systolic diameter, *SAP* Systolic arterial pressure

* = significant (*P* value < 0.05)

**Table 5:** Multiple regression final models for UAldo:C dependent variables in total population, Healthy + Stage B1 group and stage C group

	Goodness of fit	Variables in the model		
	R squared		Coefficient	Significance
Total population (a)	0.263	(constant)	−0.148	0.881
		LA/Ao	1.204	0.013*
		Sex (female vs male)	1.838	0.003*
		Chihuahua (YES vs NO)	3.615	< 0.001*
Healthy + Stage B1 (b)	0.444	(constant)	−2.537	0.101
		Sex (female vs male)	1.958	0,014*
		UP/UC	4.903	0,052
		Chihuahua (YES vs NO)	4.323	< 0.001*
		UREA mg/dL	0.089	0.018*
Stage C (c)	0.145	(constant)	2.395	0.003
		Furosemide dose (mg/kg/day)	0.336	0.024

LA/Ao: left atrium-to-aortic root ratio; sex: female = 1, male = 0; Chihuahua: YES = 1, NO = 0; *UP/UC* Urinary protein-to-creatinine ratio, *UREA* Serum urea

(a) Backward method step 0 predictors: body weight (kg), age (years), sex (female = 1 vs male = 0), Chihuahua (YES = 1 vs NO = 0), UREA mg/dL, UP/UC, LA/Ao, LVEDDn, E peak velocity m/s, spironolactone (YES = 1 vs NO = 0), pimobendan (YES = 1 vs NO = 0), furosemide (YES = 1 vs NO = 0), ACEI (YES = 1 vs NO = 0)

(b) Backward method step 0 predictors: body weight (kg), age (years), sex (female = 1 vs male = 0), Chihuahua (YES = 1 vs NO = 0), UREA mg/dL, UP/UC, LA/Ao, LVEDDn, E peak velocity m/s, STAGE (Healthy = 0, stage B1 = 1)

(c) Backward method step 0 predictors: body weight (kg), age (years), sex (female = 1 vs male = 0), Chihuahua (YES = 1 vs NO = 0), UREA mg/dL, UP/UC, LA/Ao, LVEDDn, E peak velocity m/s, spironolactone (YES = 1 vs NO = 0), furosemide dose (mg/kg/day), furosemide duration (days), ACEI dose (mg/kg/day), ACEI duration (days)

* = significant (*P* value < 0.05)

**Fig. 1:**
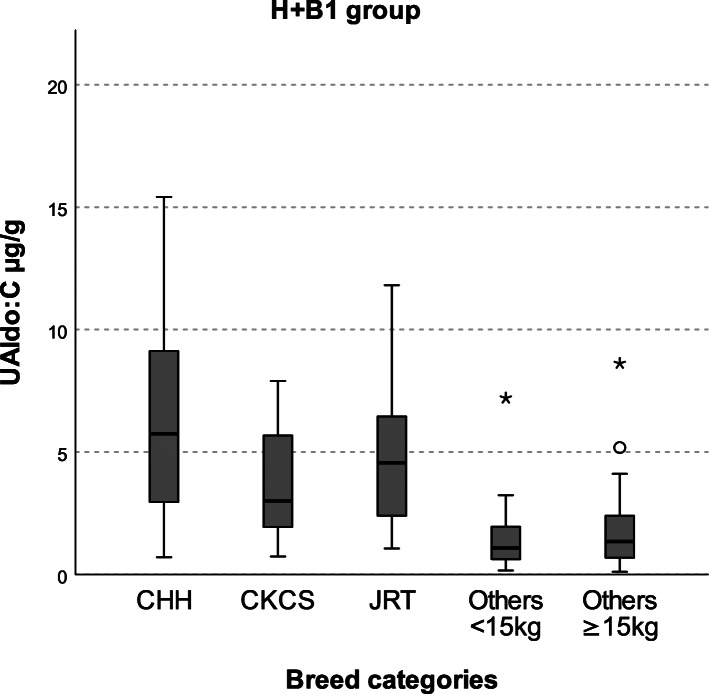
Comparison of UAldo:C among breed categories in H + B1 group. CHH: Chihuahua; CKCS: Cavalier King Charles spaniel; JRT: Jack Russell terrier; Others < 15 kg: other breeds with a body weight < 15 kg (see Table 1); Others ≥15 kg: other breeds with a body weight ≥ 15 kg (see Table 1). Chihuahua (*n* = 11; median 5.75 IQR 2.91–9.40) and CKCS (*n* = 14; median 3.00 IQR 1.90–5.70) showed significantly higher UAldo:C than others < 15 kg (*n* = 22; median 1.08 IQR 0.61–2.02) and others ≥15 kg (*n* = 40; median 1.35 IQR 0.69–2.42) (*P* values < 0.05). There were no significant differences between JRT (*n* = 7; median 4.57 IQR 2.07–7.85) and any other breed categories, as well as between others < 15 kg and others ≥15 kg

For the evaluation of correlations between UAldo:C and other variables, healthy and stage B1 dogs were grouped together (H + B1 group; *n *= 94), since they did not differ in therapy, SAP, echocardiographic measures and laboratory parameters. Comparison of UAldo:C values among breed and sex categories were performed only in H + B1 group in order to avoid any possible influence of therapy and MMVD severity. Chihuahua, Cavalier King Charles spaniel (CKCS) and Jack Russell terrier (JRT) were chosen as comparator breeds because they were the most common pure breeds in this group (as well as in the entire study population). Other breeds (see Table 1) were divided into two groups according to BW (other breeds <15 kg vs other breeds ≥15 kg). Chihuahua was chosen as the comparator breed for Pearson’s and multiple linear regression analysis since showed the highest median value of UAldo:C in H + B1 group (Fig. 1). For the linear regression analysis, the following baseline variables were considered in all analysed groups (total population, H + B1, stage C): age, BW, sex (female = 1, male = 0), breed (Chihuahua = 1, any other breeds = 0), LA/Ao, LVEDDn, E peak velocity, UREA, UP/UC. In H + B1 group, the variable STAGE (Healthy = 0, stage B1 = 1) was also included. In total population, the following variables were included in addition to the baseline ones: furosemide (YES = 1, NO = 0), ACEI (YES = 1, NO = 0), pimobendan (YES = 1, NO = 0) and spironolactone (YES = 1, NO = 0). In stage C dogs, only spironolactone (YES = 1, NO = 0) were considered, since all subjects in this stage were receiving furosemide, ACEI and pimobendan. In this group, even dosage and duration of both furosemide and ACEI were added to the regression analysis.

#### Healthy + stage B1 dogs

Urinary aldosterone-to-creatinine ratio showed a positive weak correlation with sex (female = 1, male = 0), a positive moderate correlation with breed (Chihuahua = 1, any other breeds = 0) and USG, a negative weak correlation with BW and a negative moderate correlation with age.

Chihuahua and CKCS showed a significantly (*P* values < 0.05) higher UAldo:C than other breeds < 15 kg and other breeds ≥15 kg (Fig. 1). Jack Russell terrier had numerically, but not statistically, higher UAldo:C compared to CKCS, other breeds < 15 kg and other breeds ≥15 kg.

**Fig. 2:**
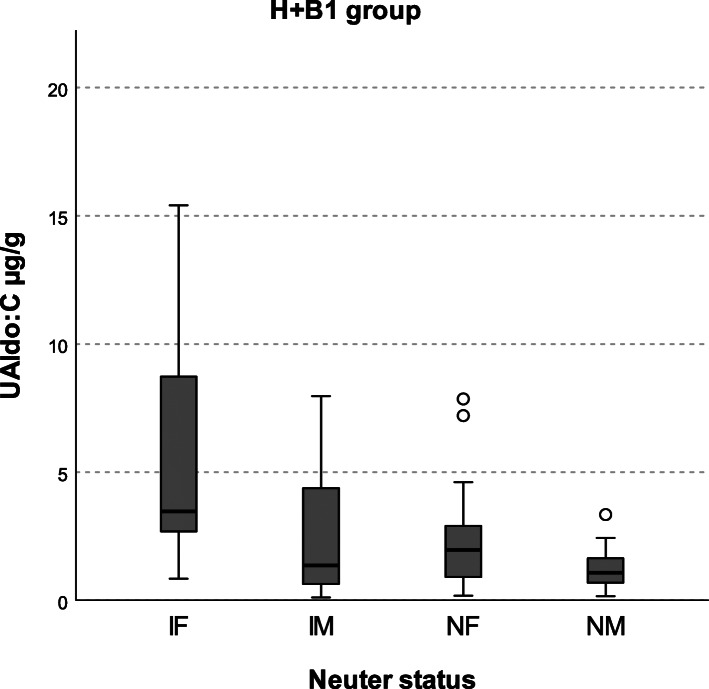
Comparison of UAldo:C among neuter status in H + B1 group. IF: intact females; IM: intact males; NF: neutered females; NM: neutered males. Intact females (*n* = 19; median 3.47 IQR 2.64–8.83) showed significantly higher UAldo:C than IM (*n* = 29; median 1.36 IQR 0.61–4.47), NF (*n* = 31; median 1.96 IQR 0.87–2.93) (*P* values < 0.05) and NM (*n* = 15; median 1.08 IQR 0.67–1.75) (*P* value ≤0.01). No significant differences were detected in UAldo:C between any other group pairs

Females showed higher UAldo:C than males (*n* = 50 vs 44; median 2.51 IQR 1.06–3.98 μg/g vs median 1.33 IQR 0.64–2.29 μg/g; *P* value < 0.05); intact females showed a significantly (*P* values < 0.05) higher UAldo:C than other neuter status (neutered females, intact males [*P* values < 0.05] and neutered males [*P* value ≤0.001]) (Fig. 2).

In the multiple linear regression analysis, UAldo:C was positively associated with sex (female = 1, male = 0), breed (Chihuahua = 1, any other breeds = 0) and UREA.

#### Stage B2 dogs

In stage B2 dogs, UAldo:C showed a moderate positive correlation with LA/Ao and positive strong correlation with UREA.

#### Stage C dogs

In stage C dogs, Pearson’s correlation did not show significant results for UAldo:C. In the multiple linear regression analysis, UAldo:C was positively associated with furosemide dose (mg/kg/day).

Within the stage C group, there was significant difference in UAldo:C between dogs treated with spironolactone (*n* = 20; 2.50 IQR 1.71–5.76 μg/g) and those not treated (*n* = 22; median 1.37 IQR 0.84–3.05 μg/g; *P* value< 0.05). These two groups were separately compared with healthy, stage B1 and stage B2 dogs, but no significant differences in UAldo:C among groups were found in both cases.

Using the median UAldo:C value of healthy dogs as cut-off (1.75 μg/g), ABT occurred in 36% (8/22) of stage C dogs not receiving spironolactone.

#### Total population

In the entire study population, UAldo:C showed a positive weak correlation with sex (female = 1, male = 0) and USG, a positive moderate correlation with breed (Chihuahua = 1, any other breeds = 0) and a negative weak correlation with age and BW.

In the multiple linear regression analysis UAldo:C was positively associated with sex (female = 1, male = 0), breed (Chihuahua = 1, any other breeds = 0) and LA/Ao.

## Discussion

Urinary aldosterone-to-creatinine ratio was not significantly different among healthy and MMVD dogs of different stages, similarly to what reported by two recent studies [17, 34]. Several factors may have influenced this result.

The RAAS activity during the asymptomatic phase is one of the most debated topics of MMVD pathophysiology since several studies have showed conflicting data [17, 27–35]. Different assays, substrates (plasma, serum, urine, tissues), RAAS components and MMVD aetiology (naturally occurring vs experimentally induced) may have contributed to the lack of univocal results. However, these conflicting data probably also reflect the heterogeneity of the pre-clinical MMVD population: the severity of the disease and the consequent hemodynamic alterations could differ widely between patients that have just developed mitral insufficiency and those near to the onset of CHF. The recent results of the DELAY study seem to support this hypothesis, since UAldo:C was found to be significantly higher in advanced stage B2 (42 months) compared to early one (day 0), showing a progressive increase over time [35]. Definitely, the number of stage B2 dogs was very low in the present study and this may have decreased the statistical power for detecting significance in differences between this group and the other ones for UAldo:C. For the same reason, the impact of pimobendan on this parameter was not evaluated. In previous studies, short-term administration of pimobendan in healthy dogs had no effect on UAldo:C [41–43]. Even in the setting of a long-term treatment, pimobendan was not significant in the multivariable model for UAldo:C in dogs with MMVD. However, data are limited to one study [17] and further investigations about chronic administration are warranted.

Stage C dogs were expected to show an overstimulation of RAAS compared to healthy and pre-clinical MMVD dogs [2, 24], but the complexity of therapy and its influence on RAAS make difficult to interpret the result. All stage C dogs were receiving drugs with opposite effects on RAAS (ACEI vs diuretic) [1, 17, 41–44] and 20 dogs were also treated with spironolactone, which prevents aldosterone’s binding to mineralcorticoid receptor, leading to an increase in blood and urine aldosterone concentrations [3, 34, 38, 45]. As expected, dose of furosemide was positively associated with UAldo:C in the regression analysis and stage C dogs receiving spironolactone showed a significantly higher UAldo:C than those not treated.

Moreover, individual factors affecting UAldo:C were found in the present study, adding further variability to this parameter.

Cavalier King Charles spaniel and Chihuahua showed a significantly higher UAldo:C than other breeds < 15 kg and other breeds ≥15 kg. In particular, Chihuahua showed the highest median UAldo:C value among breeds in H + B1 group, and in the multiple linear regression analysis this breed was positively associated with this parameter. Breed differences in RAAS activity were already reported by Pedersen et al. in 1995, which found higher plasma renin activity (PRA) and aldosterone in CKCS and Poodles compared to other breeds [46]. More recently, Hezzell et al. have found higher UAldo:C in CKCS compared to other breeds [17], while, to our knowledge, there are no reports about Chihuahua. The influence of breed on RAAS activity could be related to differences in genes encoding for RAAS components. Polymorphism of angiotensin-converting enzyme gene has been found in several breeds, including CKCS and Chihuahua, but it has been suggested that it could be more common in certain breeds [47–49]. Little is known about the effect of this polymorphism on RAAS components in dogs, but, as previously reported in humans [50], a recent study has found higher aldosterone levels and ABT incidence in MMVD dogs with ACE polymorphism after enalapril treatment and in presence of adequate ACE-A-II suppression, suggesting that this genotype could be involved in the upregulation of alternative pathways for aldosterone secretion [47]. Furthermore, polymorphism of genes encoding for angiotensinogen, A-II type 1 receptor, aldosterone synthase and chymase have been reported in humans and may also be present in dogs [51–54].

The negative association between UAldo:C and BW highlighted by Pearson’s correlation is difficult to interpret because this parameter is strictly related to breed. Parameters such as body condition score were not recorded, but we excluded clinically relevant underweight or overweight at physical examination.

In the multiple linear regression analysis, sex (female = 1, male = 0) was positively associated with UAldo:C. In H + B1 group, females showed a significantly higher UAldo:C than males and, distinguishing between neutered and intact, UAldo:C was significantly higher in intact females compared to other neuter status. To our knowledge, this is the first study that has reported an association between sex and aldosterone levels in dogs. Aldosterone levels have been showed to rise during the luteal phase of menstrual cycle in women and a relationship with progesterone concentration was found [55–57]. Progesterone showed an antimineralcorticoid effect in humans, rats and guinea pigs, binding to mineralocorticoid receptor and preventing aldosterone interaction. Thus, the physiological increase in progesterone concentration during the luteal phase likely lead to an increase in serum and urinary aldosterone levels because of the receptor occupation and the compensatory activation of RAAS [55–61]. However, it has been also reported that progesterone is able to both directly stimulate the aldosterone production from zona glomerulosa and increase adrenal sensitivity to A-II [56]. Data about estrogens are instead controversial since they have been reported to induce both an increase and decrease in RAAS components [55, 57, 61–64]. Overall, sex hormones fluctuations during menstrual/estrous cycle seem to have a significant impact on RAAS components and may have been responsible for sex differences in aldosterone levels in the present study. Thus, gender, neutering status and phase of estrous cycle should be taken into account when evaluating aldosterone levels in dogs. Sex hormones and history of estrus were not evaluated in the current study. Moreover, the interaction between gender and RAAS could be even more complex; indeed, a relationship between sex and polymorphisms of RAAS genes has been reported in people [65–68]. Thus, further investigations are needed to better elucidate the association between sex and RAAS in dogs.

Lastly, UAldo:C showed a negative weak and moderate correlation with age, although this association was lost in the linear regression analysis, suggesting that other factors (eg, breed and sex) had greater impact on this parameter. However, similar result was obtained by one previous study in dogs [17]. In people and animal models, age-related changes in RAAS were reported by several studies, which found a decline in plasma renin activity and aldosterone production with advancing age; moreover, these changes were more pronounced under stimulatory conditions, such as sodium restriction or ACTH stimulation [69–74]. With aging, normal function of many physiological systems progressively decline. Mechanisms underlying the decrease in PRA still need to be clarified, but it may be related to morphological and functional alterations of aging kidney and consequent reduction in renin activation and synthesis [69, 73, 75, 76]. The decline in aldosterone levels in older people could directly depend on lower PRA, but it has been also associated to a progressive decrease of aldosterone synthase expression [77]. Overall, older age appears to be associated with a lower physiological aldosterone secretion and with a reduced ability to respond to physiological stimuli of RAAS. For these reasons, age might contribute to the individual variability of aldosterone levels.

On the basis of the aforementioned results, a population-based reference value of UAldo:C might not be representative of the neurohormonal activity of the single patient and could lead to wrong interpretations. An individual monitoring of this parameter would likely be more accurate since it would take into account the impact of therapy and the influence of individual characteristics (ie, breed, sex and age). To our knowledge, individual monitoring of UAldo:C in MMVD dogs has been only performed in the recent DELAY study, which included only stage B2 dogs and did not aim to investigate the prognostic role of this parameter. Although the results are promising, showing an increase in UAldo:C as the disease progresses, further longitudinal studies are needed. This approach would help clarify the real diagnostic and prognostic value of UAldo:C in MMVD dogs, which might be misrepresented by mean/median values comparisons, and would help better define the ABT phenomenon. Aldosterone breakthrough occurs when aldosterone levels rise up to or above pre-treatment levels despite ACEI/angiotensin receptor blockers administration [37, 38]. In the present study, ABT was investigated in stage C dogs not receiving spironolactone using the median UAldo:C value of healthy dogs as cut-off. According to this criteria, ABT occurred in 36% (8/22) of dogs. This percentage fit well with those previously reported both in humans and dogs [37, 38]. However, this study showed that individual factors can affect UAldo:C. Thus, the use of median values might be misleading even for ABT definition. An individual monitoring before and after ACEI administration would be more accurate even for the identification of this phenomenon.

Our median normal UAldo:C value was remarkably higher than those of previous studies. Considering the aforementioned results, it is likely that breed and gender differences played an important role. In the present study, CKCS, Chihuahua, and JRT represented, together, 33% of breeds in healthy group. Females were the prevalent gender (61%), and most of them were sexually intact. In previous studies, the recruited healthy dogs were beagles or hounds (except for few crossbred) and most of them were males [40–43, 78–81]. Further studies will help confirm breed and gender inconsistencies about UAldo:C.

In the present study, LA/Ao showed a positive correlation with UAldo:C in the linear regression analysis in total population. The progression of MMVD is mediated in part by the RAAS. Impairment of cardiac function lead to RAAS activation; on the other hand, persistent high aldosterone levels contribute to cardiac remodeling through several harmful cardiovascular effects [1–7]. In humans, the association between aldosterone and left ventricular remodeling has been shown [6, 9–11]; in recent studies, aldosterone levels have also been associated with left atrial structural and functional remodeling in patients with hypertension and primary aldosteronism [12, 13]. In dogs with MMVD, UAldo:C has been positively associated with echocardiographic indicators of left ventricular remodeling [17]. In the DELAY study, treatment with spironolactone and ACEI in stage B2 dogs led to a reduction of LA/Ao and LVEDDn, confirming the negative effects of aldosterone; moreover, a progressive increase of both LA/Ao and UAldo:C during the study period (42 months) was observed in the placebo group [35]. The statistically significant association between UAldo:C and LA/Ao found in the present study support the mutual relationship between RAAS and cardiac remodeling. Left atrium dilation is probably the most important marker of MMVD progression and LA/Ao is strongly associated with time to the onset of CHF or cardiac death [82, 83]. On the basis of our result, MMVD dogs with higher UAldo:C are expected to show a more severe left atrium dilation, suggesting a possible role of aldosterone as a marker of disease progression and negative prognostic factor. In people affected by heart diseases, higher aldosterone concentrations have been associated with development of CHF and increased cardiovascular mortality [14–16], while the evidence of an influence on survival is minimal in dogs [18]. Thus, further studies in veterinary medicine are needed to explore the effect of aldosterone on outcome in patients with heart diseases.

It’s well established that aldosterone also contributes to renal damage through multiple mechanisms, such as renal hemodynamic alterations, fibrosis and oxidative stress, and has been associated with a decline in estimated glomerular filtration rate in humans [1, 4, 8, 84]. Preliminary findings in support of a relationship between aldosterone and renal function have been found in the present study: in the linear regression analysis UAldo:C was associated with serum urea. However, this association is difficult to explain as a result of aldosterone-induced renal damage since all healthy and stage B1 dogs had normal serum urea and creatinine. Veterinary medicine lacks specific studies about the pathological role of aldosterone on renal function and these preliminary findings warrant further investigations, especially in patients affected by kidney diseases.

The positive association between UAldo:C and USG observed was likely related to the sodium reabsorption and water retention induced by aldosterone.

The present study has several limitations. The first one is the low number of stage B2 dogs, which may have influenced the results obtained within this group and may have decreased the statistical power for detecting significance in differences among groups. Secondly, dietary sodium intake, time of feeding and time of urine collection were not controlled. Sodium-restriction has been associated with an increase in aldosterone levels and aldosterone has daily fluctuations, especially in relation to meals [1, 44]; thus, these aspects may have influenced UAldo:C. Thirdly, this study focused on UAldo:C, but other RAAS components also may be clinically relevant in MMVD dogs [27]. However, little is still known about their diagnostic and prognostic utility, as well as their individual variability, and further studies are needed, especially in a longitudinal setting. Definitely, a comprehensive assessment of different RAAS components should be always preferable, whenever possible, to the evaluation of a single factor in order to better characterized RAAS activity and its relation with certain variables. Lastly, aldosterone was not evaluated on other substrates, such as plasma and serum. At the current knowledge, UAldo:C seems to be the most accurate method to assess aldosterone levels in dogs [38–40]. However, further investigations about the comparison of aldosterone measurements on different substrates would be of interest.

## Conclusion

Urinary aldosterone-to-creatinine ratio was not significantly different among healthy, stage B1, stage B2 and stage C dogs. This parameter appeared to be influenced by individual factors, such as breed, sex and age, and therapy probably added further variability. This means that the use of median values of UAldo:C to interpret the RAAS activity of a single patient or of a specific MMVD stage might be misleading. An individual monitoring of this parameter may be more appropriate and would help clarify its real diagnostic and prognostic value in dogs affected by MMVD. Aldosterone breakthrough showed a prevalence of 36% in stage C dogs not receiving spironolactone and this percentage is in line with those previously reported [38]. However, due to the high individual variability of UAldo:C found in the present study, even these results should be re-evaluated in the setting of an individual longitudinal approach.

Lastly, UAldo:C was positively associated with LA/Ao, sustaining the mutual relationship between RAAS and cardiac remodeling and suggesting a possible role of UAldo:C as marker of MMVD progression.

## Methods

### Animals and study timeline

This cross-sectional study was conducted in accordance with the guidelines of the Animal Care and Use Committee of the University of Milan (approval number 2/2016) and with informed consent of the owners. All procedures to which patients have been subjected were part of their routine health screening; blood and urine analysis were performed on leftover samples. Private owned dogs were recruited among those referred to the cardiology service of the Veterinary Teaching Hospital - University of Milan, between November 2017 and December 2019.

### Inclusion and exclusion criteria

To be enrolled in the study, dogs had to be either healthy or affected by MMVD stage B1, B2 or C (ACVIM classification) [23]. No age, sex or breed restrictions were applied.

All included dogs underwent indirect blood pressure measurement, complete physical examination, echocardiography, standard urinalysis and UREA, SCr and UAldo evaluations.

A dog was considered healthy if the medical history and the results of the aforementioned procedures did not reveal any alterations. Patients that were diagnosed with MMVD by echocardiography, were classified in stage B1, stage B2 or stage C according to the criteria of the ACVIM guidelines [23]. Dogs with any cardiovascular disease other than MMVD were excluded. Subjects with clinically relevant diseases (ie, metabolic, endocrine or neoplastic) were excluded. Hypertension and chronic kidney disease were reason of exclusion only in healthy, stage B1 and stage B2 dogs. The administration of non-cardiovascular drugs with known effects on RAAS (i.e., corticosteroids) was not accepted. The administration of cardiovascular drugs was allowed for stage B2 (pimobendan) and stage C (standard therapy: furosemide, ACEI, pimobendane; ± spironolactone) dogs.

### Systolic arterial pressure measurement

Each dog was allowed to acclimate to the room for 5–10 min and SAP measurement was the first procedure to be performed based on previously published guidelines [85]. Dogs were gently restrained in ventral or lateral recumbency and SAP was measured by a Doppler sphygmomanometry method on the left thoracic limb of each dog, with a cuff size approximately 40% of the limb circumference. Blood pressure results were obtained by discarding the first measurement and averaging the following 5 consecutive ones.

The SAP measurement included in the analysis was the one recorded during the day of urine collection, in order to have temporal agreement between systemic pressure and aldosterone levels. Subjects with SAP > 160 mmHg have been re-evaluated at subsequent examinations and true hypertension was excluded in all dogs, except for 2 stage C dogs in which hypertension was confirmed.

### Echocardiography

The echocardiographic examination was performed by two experienced echocardiographers using an ultrasonographic unit (Esaote MyLab50 Gold Cardiovascular ultrasound scan) equipped with two different multifrequency phased array probes. All echocardiographic scans were carried out on conscious dogs in right and left lateral recumbency, in accordance with published standards [23, 86].

All measurements were taken from at least three consecutive cardiac cycles, and the mean was recorded. The following measurements were taken from the right parasternal short-axis view: LA/Ao measured in 2D-mode using the Hansson’s method [87], and left ventricular end-diastolic diameter and left ventricular end-systolic diameter measured in 2D-guided M-mode with the leading edge to inner edge method at the level of the papillary muscle. Normalized left ventricular end-diastolic diameter and LVESDn were obtained using the allometric equation, as previously described [88]. Transmitral flow [E peak velocity, A peak velocity, E peak velocity-to-A peak velocity ratio] was measured using continuous-wave Doppler from the left four chamber apical view.

### Sample collection, storage and analysis

All urine samples were collected by spontaneous micturition and were immediately refrigerated. Within 8 h, standard urinalysis was performed by dipstick chemistry test and refractometer (for USG evaluation); all samples were then immediately centrifugated at 1250 rpm for 5 min and supernatant was stored at − 20 °C. Supernatant underwent urinary protein and urinary creatinine evaluation by Pyrogallol Red Method and UP/UC was calculated (values < 0.5 were considered normal [89]). Samples were then submitted for determination of UAldo.

Blood samples were carried out by venipuncture at least 8 h after meal and collected into serum gel tubes. Serum urea and SCr was determined by Urease-GLDH Method and Modified Jaffe’s Method respectively (internal laboratory reference value: 20–60 mg/dL for UREA and < 1.5 mg/dL for SCr).

### Measurement of urinary aldosterone

Urinary aldosterone was determined by a commercially available species-independent ELISA kit.^2^ Before analysis, urine samples were hydrolysed to extract aldosterone metabolites using a 3-fold dilution with 0.2 N HCl and incubation in the dark at room temperature for 24 h. After the hydrolysis, the samples were diluted 30-fold in assay buffer (final dilution: 1/90) and processed immediately. The concentration of UAldo was determined following the manufacturer’s recommendations. Cross-reactivity to various steroid hormones was: 11-deoxycorticosterone 0.30%; progesterone 0.20%; corticosterone 0.19%; cortisol, dihydrotestosterone, estradiol and testosterone < 0.001%.

As the kit was developed for measuring aldosterone in human and rat samples, the accuracy of the kit for measuring aldosterone in dog urine was evaluated by recovery and parallelism studies.

In the recovery study, a pooled urine of low endogenous aldosterone was prepared by thoroughly mixing urine samples from three dogs, that was then hydrolysed as previously described. Aldosterone solution was prepared by dissolving 1 mg aldosterone (SIGMA-Aldrich, Schnelldorf, Germany) in 1 ml of 100% ethanol which was further diluted to 100 ng/mL with assay buffer. Known amounts of aldosterone (10–50-100 pg/mL) were added into the urine pooled sample and the total urine aldosterone (including endogenous aldosterone) was measured using the kit. The amount of aldosterone recovered was then calculated by subtracting the spiked dose from the value obtained for the non-spiked urine samples and the overall recovery was summarized in linear regression analysis between the measured and the added concentrations.

In the parallelism study, the slope of the standard aldosterone curve was compared with the slope of the curves obtained assaying four urine samples taken from different dogs and serially diluted in assay buffer (1/30–1/960).

Furthermore, verification of performance for precision was tested to establish that the laboratory’s performance was consistent with the manufacturer’s claims.

Precision was determined by replicate determinations of aldosterone in four urine samples with different aldosterone concentrations. Intra-assay precision was determined by evaluating each sample five times within the same run of the assay on three separate occasions. Inter-assay precision was determined by evaluating each sample in three assays on separate days. The results are reported as coefficient of variation.

Parallelism and recoveries calculations were performed with statistical methods included in the GraphPad PRISM 8.0 software package (GraphPad Software, San Diego, CA, USA).

### Statistical analysis

Statistical analysis was performed using IBM SPSS Statistics 26.

Distribution of variables was tested for normality using the Shapiro-Wilk test at the α = 0.05 level.

Normally distributed data were presented as mean ± standard deviation and compared by the two-sided Student’s t-test and non-normally distributed data were presented as median and IQR and compared by the median test; categorical data were presented as frequencies and compared by the Chi-square test.

Multiple comparisons were performed by ANOVA or median test as appropriate. Post hoc tests were performed when appropriate and Bonferroni adjusted *P* values were reported for significant findings.

Correlation was tested by the Pearson rho correlation coefficient, with the following interpretation: ≤ 0.3 weak correlation, > 0.3 and ≤ 0.7 moderate correlation, > 0.7 strong correlation.

Multiple linear regression was performed, and the backward method was used. An R square value greater than 0.1 (at least a weak correlation) at the 0.05 significance level was considered suitable. Regression was performed in the entire sample, in the group of healthy and stage B1 dogs and in stage C group, while it was not performed in stage B2 group because of low number of subjects.

A *p*-value of 0.05 was taken as statistical significance.

## Data Availability

The datasets used and/or analysed during the current study are available from the corresponding author on reasonable request.

## Abbreviations

RAAS: Renin-angiotensin-aldosterone system
MMVD: Myxomatous mitral valve disease
UAldo:C: Urinary aldosterone-to-creatinine ratio
ABT: Aldosterone breakthrough
LA/Ao: Left atrium-to-aortic root ratio
A-II: Angiotensin II
UAldo: Urinary aldosterone
ACEI: Angiotensin-converting enzyme inhibitors
CHF: Congestive heart failure
ACVIM: American College of Veterinary Internal Medicine
ACE: Angiotensin-converting enzyme
BW: Body weight
IQR: Interquartile range
SAP: Systolic arterial pressure
LVEDDn: Normalized left ventricular end-diastolic diameter
LVESDn: Normalized left ventricular end-systolic diameter
E/A: E peak velocity-to-A peak velocity ratio
UREA: Serum urea
SCr: Serum creatinine
USG: Urine specific gravity
UP/UC: Urinary protein-to-creatinine ratio
ELISA: Enzyme-linked immunosorbent assay
CKCS: Cavalier King Charles spaniels
JRT: Jack Russell terrier
PRA: Plasma renin activity
CHH: Chihuahua
IF: Intact females
IM: Intact males
NF: Neutered females
NM: Neutered males

## Footnotes

1 Enzo Life Sciences Aldosterone ELISA kit, Enzo Life Sciences Inc., Farmingdale, NY, USA

2 Enzo Life Sciences Aldosterone ELISA kit, Enzo Life Sciences Inc., Farmingdale, NY, USA
